# Waste Products Made Useful

**Published:** 1893-01

**Authors:** Lyon Playfair

**Affiliations:** North American Review


### SELECTIONS.

### Waste Products Made Useful.

BY THE RIGHT HON. LORD PLAYFAIR, F.R.S., LL.D.

As knowledge progresses, man discovers new uses for the most common objects, and learns that, though bodies may undergo many transformations, each one has its destined utility. Nature is most economical of material, and does not admit the idea that any substance can become useless. The waste matter of animals during their lives and their own bodies after death become transformed into the food of plants and constitute the basis for new generations of living beings. We know also that the very dust blown by winds from place to place has its use in the atmosphere, because, through its agency, clouds and rain are produced, as well as the glorious colors of the sky.

As nature does not admit the idea of waste matter, man, when under the guidance of knowledge, should not be inclined to deem anything as a waste product. It may be unused, because he has not learned how to apply it to a useful purpose, but the time arrives when it will be converted into a practical utility. The whole history of manufactures is a commentary on this text. The refuse of the produce of to-day may possibly become the chief source of profit to-morrow. Scarcely a single article of use or ornament, after it has served its first purpose, is not used over again for another service, perhaps in a new and distinct form, or in composition with other materials. Manufacturing industry loves to work up odds and ends and even the human refuse of our shops and homes. But these applications require a thorough knowledge of the objects to be converted into new utilities.

In the seventeenth century the illustrious Boyle wrote an essay entitled, "Man's great ignorance of the uses of Natural Things; or, that there is no one thing in Nature whereof the uses to Human Life are yet thoroughly understood." This truth of the seventeenth century is equally true as we approach the twentieth. Those who have read my articles upon air and water will recollect how slowly our knowledge accumulated in regard to these most familiar objects, and will be convinced how much remains for us in the future progress of knowledge before we or our descendants can say with truth that we really know everything about the most familiar things under our constant observation.

Lord Palmerston is credited with having invented the happy definition that "dirt is merely matter in a wrong place." It is difficult to say who is the real author of a popular apothegm. I once, in a fit of idleness, tried to trace the author of the saying that a blunt, plain speaker "calls a spade a spade." I did not succeed in finding the original author, but I found it used as a well-known proverb by Philip of Macedonia, the father of Alexander the Great. This is a parenthesis, so we may give Lord Palmerston the credit for the excellent definition of dirt, until some of my readers trace it further back. The object of this article is to show that, as science advances, it sweeps up dirt from the wrong place and deposits it in the right place.

The first illustration is the common lucifer match for producing instantaneous light or flame. This is now such an essential comfort and even necessity of our daily life that it may be difficult for some to believe it was unknown in my youth, for it was only introduced in 1833. We should remember the difficult and laborious processes employed by the ancients to obtain light and how careful they were to preserve the sacred fire when it had been procured. As the world grew older the fire-making processes were slowly improved. The *Pyxidicula Ignaria* of the Romans was probably a rude kind of tinder-box, though not in its more modern form of flint, steel, tinder and a sulphur match such as I used in my early days. It was indeed used in much the same form in the middle ages, for Philip the Good, 1429, carried the tinder-box upon the collar of the Golden Fleece, as showing to what an advanced stage of science his generation had reached. It is indeed surprising that phosphorus matches were so slowly discovered, for the element of phosphorus had been described by an Arabian called Bechel in the eighth century, though it was forgotten, and had to be rediscovered by Brandt in 1669. Both of them got it out of liquid human refuse, after it had been changed by keeping. Subsequently a cheaper and less repulsive raw material was found in old bones, which are rich in phosphate of lime; now the skeletons of carcasses are chiefly used for the preparation of lucifer matches. Though phosphorus was known and its remarkable properties had become familiar, it was not till 1833 that the first phosphorus friction match was invented. I remember my own extreme joy when the phosphorus match was first invented. Intolerably bad the first matches were, dangerously inflammable, horribly poisonous to the makers and injurious to the lungs of the users. It was not till 1845 that my friend Schroter, of Vienna, showed that by heating phosphorus in an oil bath it became converted into the form of red brick, which was an allotropic condition of the element which did not poison the makers of matches and was much less inflammable than ordinary phosphorus.

What a wonderful change has been produced in all our habits by the ready means of obtaining light out of the material formerly extracted from human effete matter, and now from old bones! Before its application to matches it is calculated that every man, woman and child spent ninety hours yearly in getting light and fire, or rather that they would have done so if they had used such means as freely as we do now. At present the consumption of phosphorus matches per head of the population amounts to eight daily, and as each match consumes fifteen seconds in its use two minutes are spent for the whole day, or twelve hours for the year. If we calculate the economy of time to the population of the United States by this simple invention, each person saves seventy-eight hours yearly; or, say, ten working days, which, represented in labor, cost at half a dollar per day for the sixty-two-millions of the population in the United States, gives an aggregate economy of three hundred and ten million dollars yearly.

Originally, phosphorus was made from liquid effete matter of human beings, and, unhappily, that is treated to a great extent as waste even now, for it is allowed to run into the sea by the drains. Yet every pound of it, if properly applied in agriculture, is capable of producing a pound of wheat. Victor Hugo, who early wrote on such subjects, was moved to an indignant protest in "Les Miserables:"

"Science, after long experiment, now knows that the most effective of manures is that of man. The Chinese, we must say to our shame, knew it before us. No Chinese peasant, Eckberg tells us, goes to the city without carrying back, at the two ends of his bamboo, two buckets full of what we call filth. Thanks to human fertilization, the earth in China is still as young as in the days of Abraham. Chinese wheat yields one hundred and fifty fold. To employ the city to enrich the plain would be a sure success. If our gold is filth, on the other hand our filth is gold. What is done with this filth which is gold? It is swept into the abyss."

The agriculture of the United States is an industry great in its extent, but it is probably the most thriftless industry in existence. English agriculture is bad enough, but it produces on an average from thirty to forty bushels of wheat per acre. But the farmers of the United States produce an average of only twelve bushels per acre. This arises from the temptation to seek new land when the old farms have become unproductive from a wasteful system of agriculture.

In the year 1842 I had the pleasure to accompany the illustrious German chemist, Baron Liebig, in a tour through Great Britain. On one of our excursions, my friend, the eminent geologist, Dr. Buckland, joined us, and he took us to see some curious concretions in rocks of Tertiary formations. Buckland had for some time suspected that these stone nodules really were the fossils of the dung of ancient Saurian reptiles, which dwelt on the earth long before man's appearance upon it. As a proof, he showed that the concretions had a spiral twisting like that seen in the exuviæ of living fishes. Liebig suggested that it would be a better proof of fossil dung if chemical analysis showed that it was rich in phosphates. I sent a portion of it to my laboratory, and Liebig's belief was confirmed, as all the concretions were rich in bone earth. The name *coprolite* was given to this fossil dung (*kopros*, dung, and *lithos*, a stone). The publication of this discovery led to the establishment of extensive industries for the preparation of superphosphates as manure, although now the use of coprolites is being superseded by the discovery of mineral phosphates in rocks. Besides its use as manure, human refuse is still largely employed in making ammonia and its salts, which are largely used in the industrial arts, in agriculture, and in medicine. Originally, *sal ammoniac* was prepared from the soot of the burnt sacrifices at the temple of Jupiter Ammon, from which the word ammonia is derived. At a later period it was obtained from camel's dung, and then for many centuries out of human refuse. It is even now made from that, for two thousand two hundred tons are taken daily out of the cesspools of Paris to be converted into ammonia. Luckily the Parisian ladies, when they use their scent bottles, little suspect the origin of the pungent odor. Ammonia, is however, made on a more extensive scale from the refuse of gas works. The users of Morocco leather little suspect that the goat skins converted into it have been liberally treated with the sweepings of dog kennels.

Many kinds of waste materials of certain manufactures are employed in new forms for other industries. Old rags are a familiar instance of this change. Cotton and linen rags form the chief raw material for the paper maker. Even those, which a beggar would disdain to touch, are converted into the paper used to convey our sentiments of love and friendship. Baron Liebig endeavored to find some material, which, by its use among various nations, would form an index to their relative degree of civilization. He fixed upon soap as showing the cleanliness or filth common to the people. The average consumption of soap gave the scale of civilization. I am more inclined to consider that the competition for cotton and linen rags denotes a still better index, because it is a measure of the distribution of education and love for literature. I have no statistics since 1887, but in that year the proportion of paper used in different countries was 12 lbs. per head in the United Kingdom; 10 lbs. in the United States; 9 lbs. in Germany; 8 lbs. in France, and 4 lbs. in Italy. The mother country and the United States are thus in the lead.

Woolen rags are more slowly converted into final products than those of cotton and linen, because they are valuable for intermediate uses. Before they are run to earth they do duty for many forms of cheap clothing. In the United Kingdom Batley, Dewsbury and Leeds are the grand markets for woolen rags, though the United States are running us in close competition. The greasy, frowsy, cast-off cloths of Europe reappear in pilot cloths, Petershams, Beavers, Talmas, Chesterfields and Mohairs, which modern dandies wear when they consult economy as well as their outward appearance. Shoddy and mungo, the resurrection raw material of greasy beggars, mixed with a varying amount of true wool, is supposed to constitute about one-third of the woolen manufactures. This raw material for adulteration is, however, only made from rags which have already served higher purposes before this tertiary use. When woolen rags still adhere together they first go through the hands of various artists, who are named "clobberers," "revivers," and "translators." The function of the clobberer is to patch up torn garments and restore them to their pristine appearance. The reviver rejuvenates seedy black coats, and sells them to customers seeking for cheap garments. The translator is an artist of a higher order, for he transforms the skirts of old coats into waistcoats and tunics for children. When black coats are too far gone to be clobbered or revived, they are sent to various countries to be made into caps, France, Russia, and Poland requiring them in large quantity. The worn-out red tunics of British soldiers almost exclusively go to Holland to cover the chests of sturdy Dutchmen, who conceive them to be a protection against rheumatism. Uniforms of a better description, whether military or liveries, chiefly go to Africa for the wear of kings and chiefs. It is only after these transformations that the rags are torn down into shoddy and mungo for inferior cloths.

When old woolen rags have reached their fourth stage of degradation, so that they are unfit for the shoddy maker, they are still economically useful. They are then mixed with other degraded waste, such as shavings of hoofs and horns, and the blood of slaughter houses, and are melted in an iron pot with wood ashes and scrap iron. This process produces the material out of which the beautiful dye, Prussian blue is made.

I fancy that I have convinced my readers, with perhaps some shock to their sentiment, that dirt is merely matter in a wrong place. When converted into an utility it is no longer dirt, for it has been purified. Manufacturers are only imitating Nature in these transformations. It may be disagreeable to sentiment, but it is strictly true, that our daily food contains the materials of previous generations of living animals, including the human race.

As to perfumes, there are some which are really oils and ethers extracted from flowers. There are others which are made artificially, and curiously, most frequently, out of bad smelling compounds. The fusel-oil, separated out in the distillation of spirits, has a peculiarly nasty and sickening odor. It is used, after treatment with acids and oxidizing agents, to make the oil of apples and the oil of pears. Oil of grapes and oil of cognac are little more than fusel-oil largely diluted. Oil of pineapple, on the other hand, is best made by the action of putrid cheese on sugar, or by distilling rancid butter with alcohol and oil of vitriol. This oil is largely used for making pineapple ale. Many a fair forehead used to be damped with "Eau de Millefleurs" without knowing that its essential ingredient was got from the drainings of cow houses, though now it can be obtained cheaper from one of the constituents of gas tar. Out of the latter is got oil of bitter almonds, so largely used to perfume soap and confectionery.

This leads me to refer to gas tar, once the most inconvenient of waste materials. It could not be thrown away into rivers, for it polluted them foully. It could not be buried in the earth because it destoyed vegetation all around. In fact nothing could be done with it except to burn or to mix it with coal as a fuel. Gas tar, formerly the most useless of waste substances, is now the raw material for producing beautiful dyes, some of our most valued medicines, a saccharine substance three hundred times sweeter than sugar, and the best disinfectants for the destruction of germs of disease. Tar has become so prolific in useful industries that it would take a long article to describe them in detail, so I can only allude to a few of them. There are two substances in tar called naphthalene and anthracene. The former of these was a waste material, which choked gas pipes and was particularly obnoxious in gas works. Every ounce of it is now of value for the preparation of dye stuffs, as is also anthracene, a body which distils over when the tar oils have got a boiling point above 300°.

Perhaps the most important use is in the manufacture of alizarin, the coloring matter found in the root of the madder plant, so extensively used at one time in making Turkey reds and in calico printing. The discovery of its artificial preparation from the waste products of tar has destroyed a great agricultural industry which flourished in Turkey, Holland, Alsace, and other countries. Not only the red dye stuff alizarin, but also beautiful blue and purple dyes are made out of the same substances.

There is another product called aniline, which exists naturally in coal tar, but can also be made in large quantities out of another substance named benzine, after it has been acted on by nitric acid and then by iron filings Aniline has become a most productive source of coloring matter, and many of its derivations are familiarly known under the names of mauve, magenta, uraniline, and other dyes. They are too numerous to describe, but there is scarcely a shade of color which cannot be obtained from some of the products of tar. Large manufactories are in existence, some of which contain forty or fifty trained chemists engaged in superintending operations, or in making researches for new coloring materials. The whole of the great industries of dyeing and calico printing has been revolutionized by the new coloring matters obtained from the old waste material—gas tar. By a very interesting series of transformations one of the constituents of coal tar has been changed into the coloring matter of indigo. Hitherto the cost of production of artificial indigo has been too great to allow it to take the place of natural indigo, the cultivation of which is one of the staple industries of the East Indies. But its cultivators tremble lest they should find themselves in the position of the growers of madder by a cheap artificial production of indigo blue from coal tar.

I have noticed in American newspapers that many cases of arsenic poisoning have been observed in the United States, produced by dresses or articles of furniture. Coal tar colors are often made with arsenic, and the danger is not overrated.

When Bishop Berkeley wrote his famous treatise on tar water, claiming it as a universal medicine, curing all diseases, he little dreamt that the time would arrive when beautiful medicinal preparations would be made out of it.

As a fact, important narcotics and febrifuges have forced their way into medicine from this source, and are much valued by physicians. The most curious of the useful products of coal tar is saccharine, a substance so sweet that the sensation on the palate is disagreeable from its cloying persistency. A grain or two grains give the sweetness of one or two lumps of sugar, and it can be taken in food without producing the dyspeptic and gouty results which real sugar produces on some persons. Thus one of the most hopeless forms of waste matter—tar—has, by our better knowledge, become productive of great uses to mankind.

I shall content myself with only one further illustration.

Of all living things rats seem to be among the most repulsive; and when dead what can be their use? But even they are the subjects of production in the industrial arts. In Paris there is a pound surrounded by walls into which all dead carcasses are thrown. A large colony of rats has been introduced from the catacombs. The rats are most useful in clearing the flesh from the bones, leaving a clean-polished skeleton fitted for the makers of phosphorus. At the base of the wall numerous shallow holes are scooped out just sufficient to contain the body of the rats but not their tails. Every three months a great *battue* takes place, during which the terrified rats run into the holes. Persons go round and catching the extending tails, pitch the rats into bags, and they are killed at leisure. Then begins the manufacture. The fur is valuable and finds a ready sale. The skins make a superior glove—the *gant de rat*—and are especially used for the thumbs of kid gloves, because the skin of the rat is strong and elastic. The thigh-bones were formerly valued as tooth-picks for clubs, but are now out of fashion; while the tendons and bones are boiled up to make the gelatine wrappers for bon-bons.

Surely I have established my thesis that dirt is only matter in a wrong place.

Chemistry, like a thrifty housewife, economizes every scrap. The horseshoe nails dropped in the streets are carefully collected, and reappear as swords and guns. The main ingredient of the ink with which I now write was probably once the broken hoop of an old beer barrel. The chippings of the travelling tinker are mixed with the parings of horses' hoofs and the worst kinds of woolen rags, and these are worked up into an exquisite blue dye, which graces the dress of courtly dames. The dregs of port wine, carefully decanted by the toper, are taken in the morning as a seidlitz powder, to remove the effect of the debauch. The offal of the streets and the wastings of coal gas reappear carefully preserved in the lady's smelling bottle, or used by her to flavor blanc manges for her friends. All this thrift of material is an imitation of the economy of nature, which allows no waste. Everything has its destined place in the process of the universe, in which there is not a blade of grass or even a microbe too much, if we possessed the knowledge to apply them to their fitting purposes. Man aims at the acquisition of this knowledge, and, as we attain it, we are always rewarded by indirect though important benefits to the human race. It is neither necessary nor desirable that we should seek knowledge for the sake of utilities: our reward comes when we search for truth, because it is truth. If we try to use the rays of knowledge on account of their own inherent beauty, their reflection upon all things, animate and inanimate, show properties of matter which range themselves into utilities, almost without our perceiving the process, and teach us that there is nothing common or unclean to the laws of science.

LYON PLAYFAIR.

*North American Review.*

---

THE temperance women of Chicago have the courage of their convictions anyhow. An offer of $1,500 a month rent was made to them for the right to put a gilded palace, in which to sell tobacco, in the rotunda of their marble halls in the Temperance temple. But Mrs. Carse answered: "Admit the vile weed into our temple of truth and purity? Never!"

